# Phylogeographic investigation and ecological niche modelling of the endemic frog species *Nanorana pleskei* revealed multiple refugia in the eastern Tibetan Plateau

**DOI:** 10.7717/peerj.3770

**Published:** 2017-09-11

**Authors:** Bin Wang, Feng Xie, Jiannan Li, Gang Wang, Cheng Li, Jianping Jiang

**Affiliations:** 1 CAS Key Laboratory of Mountain Ecological Restoration and Bioresource Utilization, Ecological Restoration and Biodiversity Conservation Key Laboratory of Sichuan Province, Chengdu Institute of Biology, Chinese Academy of Sciences, Chengdu, Sichuan, China; 2 Nanjing Institute of Environmental Sciences Under Ministry of Environmental Protection, Nanjing, Jiangsu, China

**Keywords:** Phylogeography, Tibetan Plateau, Species distribution modelling, Demography, Genetic structure, Multiple refugia

## Abstract

The largest plateau Tibetan Plateau supplied an excellent opportunity to investigate the influence of the Pleistocene events on the high-elevation species. To test for the alternative hypotheses of Pleistocene glacial refugia, we used partial sequences of two mitochondrial genes and one nuclear gene to examine the phylogeographic patterns of the endemic frog species *Nanorana pleskei* across its known range in the eastern Tibetan Plateau, and conducted species distribution modelling (SDM) to explore changes of its distribution range through current and paleo periods. In all data sets, the species was divided into lineage north occupying open plateau platform and lineage south colonizing the mountainous plateau. The divergence of two major clades was estimated at the early Pleistocene. In mtDNA, lineage north contained northeastern and northwestern sublineages, and lineage south had two overlapping-distributed sublineages. Different lineages possessed distinct demographic characteristics, i.e., subdivision in the northeastern sublineage, historical bottleneck effects and recent expansions in the northwestern sublineage and the southeastern sublineage. SDMs depicted that stable suitable habitats had existed in the upper-middle streams of the Yellow River, Dadu River, Jinsha River and Yalong River. These regions were also recognized as the ancestral areas of different lineages. In conclusion, *Nanorana pleskei* lineages have probably experienced long-term separations. Stable suitable habitats existing in upper-middle streams of major rivers on the eastern Tibetan Plateau and distinct demographic dynamics of different lineages indicated that the lineages possessed independent evolutionary processes in multiple glacial refugia. The findings verified the profound effects of Pleistocene climatic fluctuations on the plateau endemic species.

## Introduction

Pleistocene climate fluctuations are believed to cause many temperate species to change distribution range and evolutionary history (Hewitt, 2000, 2004). Allopatric populations in different regions might be glacially isolated and experienced postglacial expansions (Galbreath, Hafner & Zamudio, 2009; Wang et al., 2013). The isolation level contributed to substantial genetic heterogeneity, and high spatial connectivity might be in favor of gene flow and as well, in these processes, demographic dynamics of lineages presented correspondent tendency (Stewart et al., 2010; Peterson & Ammann, 2013). The models have been extensively investigated in Europe and North America (Hewitt, 2004). In East Asia, the issues have been gradually disclosed with increasing phylogeographic researches (Qu et al., 2010; Liu et al., 2013, 2015), but that is far from well understanding them especially in plateau regions.

Glaciations on Asian plateaus were considered to be asynchronous, even within regional mountain-plateau regions (Owen, Finkel & Caffee, 2002; Owen et al., 2008; Thompson, Thompson & Davis, 2006). As the highest and largest plateau on Earth, the Tibetan Plateau extends several million square kilometers and carries area of an average elevation of 4,500 m above sea level (m.a.s.l). Historical uplifts of the plateau promoted geological and climatic transformations in interior basins and large mountains around it (An et al., 2001). During the Pleistocene, different-periods and different-regions glaciations were indicated by increasing evidences (Shi, 2002; Zheng, Xu & Shen, 2002; Owen et al., 2005). Moreover, this region carries habitats that are nearly unique for Oriental and Palearctic organisms that occur in temperate ecosystems and are inhabited by considerable high-elevation endemic species (Zhang, 2009; Myers et al., 2000).

So the Tibetan Plateau supplied a fine biogeographical condition for investigating the influences of Pleistocene climatic oscillations on the evolutionary history of the high-altitude species. Some investigations have focused on species endemic to plateau and surrounding mountains (Cun & Wang, 2010; Hofmann, 2012; Zhou et al., 2013), but conflicting indications were proposed from patterns of different species. The model of refugia-in-periphery has been suggested by studies on several plant and animal species occupying the Tibetan Plateau and peripheral mountains (Frenzel, Bräuning & Adamczyk, 2003; Zhang et al., 2005; Yang et al., 2008). Recently, many works have indicated that multiple refugia exist in the plateau interior (Liu et al., 2015; Hofmann, 2012; Wang et al., 2010). Therefore, more investigations especially plateau endemic species are needed to reveal the historical scenarios.

Amphibians are considered to be indicators of climate changes because their high sensitivity to environmental changes (Bossuyt & Milinkovitch, 2001; Heinicke, Duellman & Hedges, 2007; Fouquet et al., 2012) and their physiological constraints. A small number of anuran species were endemic to high-elevation Tibetan plateau and among them *Nanorana pleskei* are distributed from 3,300 to 4,500 m.a.s.l, occupying most Hengduan Mountains and eastern Tibetan Plateau (Fei et al., 2009). The distributional range of the species almost covers origin area of many important river systems, i.e., Yellow River, Dadu River, Minjiang River, Yalong River, Jinsha River and Lancang River, and this region present terrain of open plateau platform in the northwest and mountainous plateau in the southeast (Fig. S1). Obviously, the distributional patterns of the species had been certainly influenced by the Pleistocene climate changes, and it is a striking question whether the species had refugia-in-periphery or multiple-refugia-in-plateau during the glacial epoch.

To test the hypotheses, in this study, we investigated the phylogeographic structure and ecological niche modelling of *Nanorana pleskei*. We sequenced two mitochondrial genes and one nuclear gene of sampling populations across the distribution range of the species. We aimed to: (1) examine the genetic diversity and genetic structure of the species; (2) explore the characteristics of historical demography; (3) explore whether the species retreated to a narrow refugium at the edge of the plateau or occupied multiple refugia on the plateau during the glacial periods. Integrative implements of phylogeographic analyses and species distribution modelling (SDM) allow us to explore architecture of the evolutionary history and demography dynamics of the plateau endemic species.

## Methods

### Sampling and sequencing

**Figure 1:**
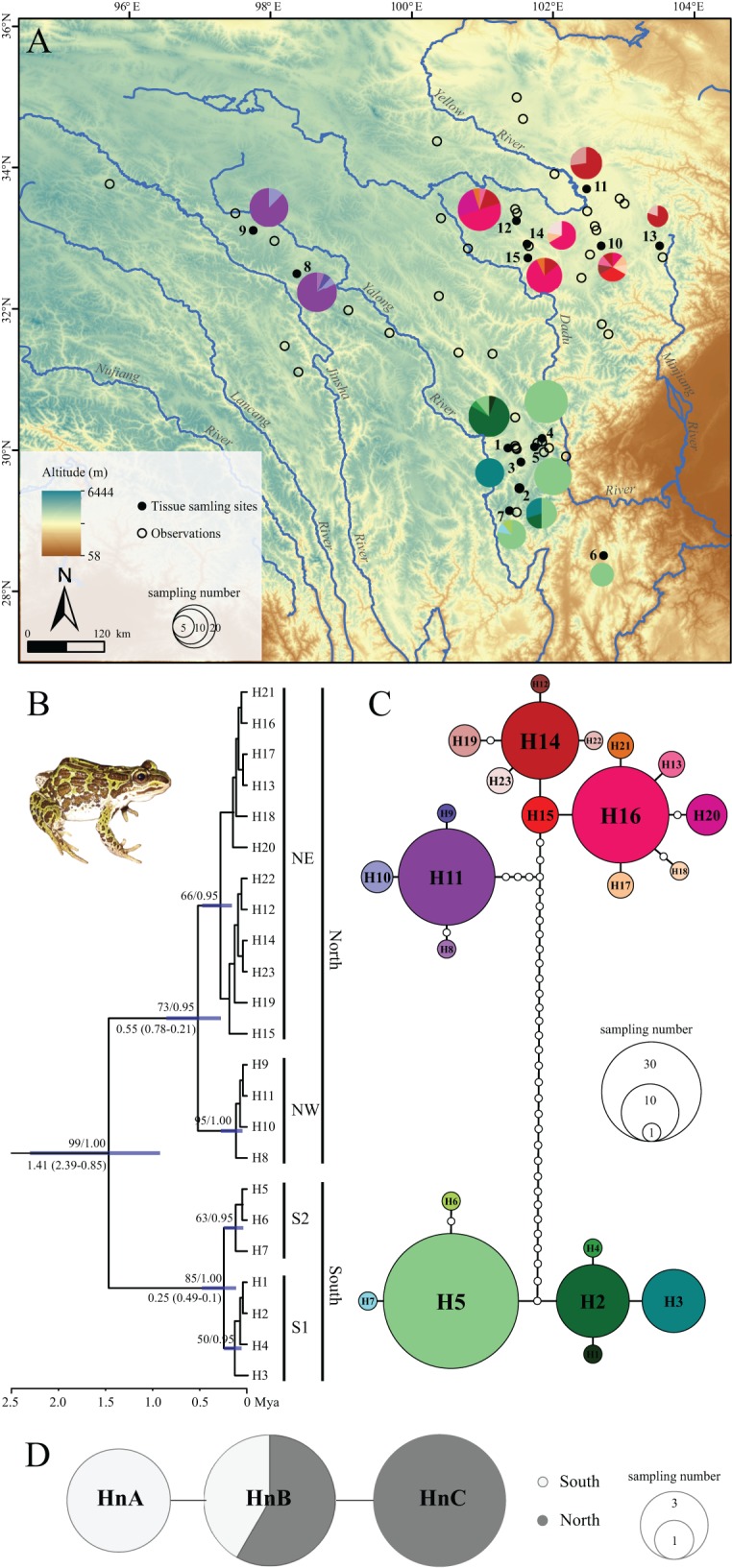
Sampling localities, phylogenetic tree and TCS haplotype network for *Nanorana pleskei*. (A) Sampling localities. The pies represent the haplotype (H1–H23; colors for each haplotype refer to C haplotype network) frequency in each population. (B) Phyloengetic tree. Maximum likelihood bootstrap support values/Bayesian posterior probabilities are above the branches. Mean time (Mya) to the most recent common ancestor (TMRCA) with 95% highest posterior density (95% HPD) for the key nodes are given below the relative branches. (C) Haplotype network for mitochondrial DNA data. Colors in the haplotype network represent different haplotypes. Sizes of cycles indicate the haplotype frequencies. Network branches linking the cycles indicate one mutation step; more mutations are represented by dark spots crossed with the branches. (D) Haplotype network for nuclear DNA data. MtDNA lineage South was offwhite, and mtDNA lineage North was dark grey.

A total of 188 individuals were collected from 15 localities mainly spanning across the known range of *Nanorana pleskei* on the eastern Tibetan Plateau (Table S1; Fig. 1A). Tissues were preserved in 95% Ethanol at −20 °C until DNA extractions were performed. For the phylogenetic analyses, two individuals of the closely-related species *Nanorana ventripunctata* were collected, and one individual of congeneric species *Nanorana parkeri* was also included as an outgroup according to previous phylogenetic analyses (Jiang et al., 2005; Che et al., 2010). The Animal Care and Use Committee of Chengdu Institute of Biology, CAS provided full approval for this purely observational research (Number: CIB2014031010). Field experiments were approved by the Management Office of the Zoige Nature Reserve (project number: ZNR201303006).

Total genomic DNA was extracted using a standard phenol–chloroform extraction procedure (Hillis et al., 1996). Two mithochondrial DNA (mtDNA) fragments, the cytochrome oxidase subunit I (COI) and the NADH dehydrogenase subunit 1 (ND1), were amplified for all samples, and fragments of one single-copy nuclear genes, the exon 1 of rhodopsin (Rhod) gene, were amplified for 30 specimens interspersing in major mtDNA lineages (see “Results”). Primers for amplifying COI and ND1 genes were newly designed using the Primer-BLAST tool on NCBI web with default settings. The complete mitochondrial genome of *Nanorana pleskei* (GenBank Accession no.: HQ324232.1) was downloaded as template for primer-designing. Primer information was shown in Table S2. Amplifications of mtDNA fragments were performed under the following conditions: 95 °C for 3 min; 35 cycles of 95 °C for 35 s, 53 °C (for ND1)/58 °C (for COI) for 35 s, and 72 °C for 70 s; and 72 °C for 10 min. Amplification of the Rhod gene was performed according to the procedures and using primers in the previous studies (Che et al., 2009). PCR products were sequenced in an ABI 3730 sequencer in both directions.

Mithochondrial DNA sequences were assembled in SEQMAN v7.21 (DNAStar Inc., Madison, WI, USA) and aligned using CLUSTAL X v1.83 (Thompson, Gibson & Plewnia, 1997) with default settings and manually checked. Two mtDNA fragments were concatenated and haplotypes were determined using DNASP v5.10.1 (Librado & Rozas, 2009). For the Rhod gene, recombination detection was conducted using seven methods in the program RDP v4.56 (Martin et al., 2015). No signal was found for recombination. Haplotypic state of the Rhod gene was inferred under a Bayesian framework in PHASE (Stephens & Donnelly, 2003) with a probability threshold of 90% and five independent runs. The input files for PHASE were produced by the web program SEQPHASE (http://seqphase.mpg.de/seqphase/). All sequences were submitted to GenBank with accession numbers KX806663, KX806664–KX806851, KX806852–KX807039, KX807040–KX807069, KX807070, KX807071–KX807072.

### Phylogenetic inferences and dating estimations

Because there were few variable sites in nuclear gene dataset (only three haplotypes were identified in all 30 sequences of the Rhod gene; see “Results”), phylogenetic analyses were conducted only based on mtDNA dataset. MtDNA data were analyzed using maximum likelihood (ML) and Bayesian inference (BI) methods, as implemented in the program PHYML 3.0 (Guindon & Gascuel, 2003) and MRBAYES v3.2.5 (Ronquist et al., 2012), respectively. Before ML and BI analyses, we divided each data set into six partitions through defining the first, second and third codon positions of protein-coding genes, and then used PARTITIONFINDER v1.1.1 (Lanfear et al., 2012) to select best-fitting partitioning schemes and the best-fitting nucleotide substitution model of each partition under the Bayesian information criterion (Schwarz, 1978). The PARTITIONFINDER analysis selected the K80 model for (COI_pos1 + ND1_pos1), F81+I model for (COI_pos2 + ND1_pos2) and TrN model for (COI_pos3 + ND1_pos3). ML and BI analyses were conducted under the selected best-fitting partitioning schemes and the best-fitting nucleotide substitution model of each partition. For ML analyses, the default tree search approach using simultaneous nearest neighbor interchange method and BioNJ tree as starting tree was used to estimate ML tree topologies. Non-parametric bootstrapping with heuristic searches of 1,000 replicates was used to assess confidences of branches in ML trees (Felsenstein, 1985; Felsenstein & Kishino, 1993; Huelsenbeck & Hillis, 1993). For BI analyses, we unlinked parameters for each partition and allowed branch lengths to vary proportionately across partitions. Two independent runs were initiated each with four simultaneous Markov Chain Monte Carlo (MCMC) chains for 30 million generations and sampled every 1,000 generations. Convergence of runs and burn-in period (first 25%) was determined using the program TRACER v1.6 (Rambaut et al., 2014a). A final majority-rule BI tree and the posterior probabilities were achieved from the remaining trees.

Based on mtDNA data, the time to the most recent common ancestor (TMRCA) of *Nanorana pleskei* was estimated using a Bayesian MCMC approach under a “relaxed molecular clock” model in BEAST v1.8.1 (Drummond et al., 2012). There was no fossil record of *Nanorana pleskei* that could be used for calibrations. The mutation rate of mitochondrial genes has been found to be broadly constant at 0.57–0.96% change per lineage per million years across many amphibian groups, such as hynobiid salamanders (Weisrock et al., 2001), *Bufo* (Macey et al., 1998), Ranid frogs (Macey et al., 2001) and *Eleutherodactylus* toads (Crawford, 2003). The *Bufo* species were indicated to have a broadly universal substitution rate of 0.65% change per lineage per million years on the mitochondrial ND1–ND2 gene region (Macey et al., 1998), while the *Rana boylii* species group was also suggested to have a broadly universal substitution rate of 0.65% change per lineage per million years on the mitochondrial ND1, ND2 and CO1 genes (Macey et al., 2001). Accordingly, here we used this mean rate to estimate divergence time among *Nanorana pleskei* lineages. In BEAST analyses, no partition scheme was selected because we used one mean rate for whole mtDNA data. The GTR + G + I nucleotide substitution model, the most flexible model available in beast, was used to allow the sample space of the parameters to be fully exploited (Huelsenbeck & Rannala, 2004). Genealogy was reconstructed with an uncorrelated lognormal tree prior with a constant population size assumption. A lognormal mean substitution rate of 0.65% change per lineage per million years was used with a lognormal standard deviation of 0.7 producing a 95% credible sampling interval (CI) from 0.5% to 1% change per lineage per million years. The MCMC chains were run for 50 million generations with sampling every 1,000 generations and 10% of the initial samples were discarded as burn-in. The convergence of chains was verified using Tracer by checking that the sampling achieved stationarity and the effective sample size (ESS) for parameters sampled from the MCMC analyses was more than 200. The remaining trees were used to obtain the subsequent maximum clade credibility summary tree with posterior probabilities using TREEANNOTATOR (Rambaut et al., 2014b).

### Population genetic structure and demography analyses

Haplotype diversity (*Hd*) and nucleotide diversity (π) for mtDNA lineages and populations were estimated using DNASP. The significance was tested using 1,000 computer permutations. To visualize geographical patterns of genetic diversity, *Hd* and π were spatially interpolated using the Kriging method, implemented in the “Geostatistical Analyst” of ARCGIS v10.2 (ESRI, Redlands, CA, USA).

Because the nuclear sequences contain few variable sites and include only a subset of specimens, the relationships between haplotypes of the nuclear gene were only shown using haplotype network. Haplotype networks of mtDNA and the Rhod genes were constructed using maximum parsimony method in the software TCS v1.21 (Clement, Posada & Crandall, 2000).

To access the extent to which the phylo-groups and/or geo-groups explain variation, hierarchical analyses of molecular variance (AMOVA) was conducted in ARLEQUIN v3.1 (Excoffier, Laval & Schneider, 2005). Three kinds of population arrangements were tested: two major lineages, four sublineages and three geographical groups (see “Results”). The significance of the fixation indices *F*ct, *F*sc and *F*st derived from AMOVA analyses was determined by 1,000 permutation replicates.

Pairwise *F*st between lineages and populations was calculated in ARLEQUIN. To test whether genetic differentiation between populations was derived from the isolation by distance (IBD) model with a significant correlation between geographic and genetic distance (*F*st), Mantel tests with 10,000 randomizations were performed in the program IBDWS v3.23 (Jensen, Bohonak & Kelley, 2005).

Genetic signals of departure from neutrality (potential population expansion) were estimated using Tajima’s *D* (Tajima, 1989) and Fu’ *Fs* (Fu, 1997) statistics. The values were calculated in Arlequin with 1,000 computer permutations. Mismatch distributions comparing observed and expected distributions of pairwise nucleotide differences were also simulated to test a model of a sudden population expansion. The significance of deviation from this model was evaluated using sum of squares deviations (SSD) and Harpending’s raggedness index (HRI) with significant *P* values indicating rejection of the recent expansion hypothesis. The analyses were implemented in ARLEQUIN and the significance of deviation of values was tested with 10,000 permutations. The coalescent-based Bayesian skyline plot (BSP), as implemented in BEAST, was used to exhibit demographical fluctuations. The analyses were only conducted for total population and two major lineages because coalescent simulations of sublineages could not reach convergence due to much less variations and samples. In BSP analyses, the starting tree was randomly generated and Bayesian skyline was used for the tree prior. According to the number of samples, two to four grouped coalescent intervals were prior chosen for lineages. The lognormal relaxed clock model and the mutation rate of 0.65%/Ma (95% CI’s: 0.5–1.0%/Ma) as suggested above were specified. MCMC chains were run for 100 million iterations with sampling every 1,000 iterations and discarding the first 10% as burn-in. TRACER was used to check for convergence of chains and ESS.

### Biogeographical analyses

The SDM was inferred using maximum entropy machine-learning algorithm in MAXENT v3.3.3k (Phillips, Anderson & Schapire, 2006). Nineteen bioclimatic layers (BIO1-19) describing temperature and precipitation were downloaded from the WorldClim v1.4 database (http://www.worldclim.org) (Hijmans et al., 2005). The layers of the last glacial maximum (LGM, ∼21,000 years before present) scenarios were obtained by the community climate system model (CCSM; 2.5 arc-minutes resolution; Collins et al., 2006) and the model for interdisciplinary research on climate (MIROC; 2.5 arc-minutes resolution; Hasumi & Emori, 2004). The layers of the current (1950–2000) and last interglacial (LIG; 140,000–120,000 years before present) conditions were retrieved with a resolution of 30 arc-second (Otto-Bliesner et al., 2006), and then were resampled into 2.5 arc-minutes resolution. To eliminate the effect of model over-fitting, Pearson’s correlation coefficient (*r*; Pearson, 1895) were used to evaluate pairwise correlations between bioclimatic variables. When the absolute value of *r* > 0.9 indicating that two variables were highly correlated, the one that was more biologically meaningful for the species was chosen for analyses. The results suggested that seven variables (BIO1-4, 12, 14 and 15; see www.worldclim.org/bioclim) could be chosen for SDMs. Correlation structure among these bioclimatic variables appeared to be stable and consistent across time periods (Mantel test: *P* < 0.01), suggesting that the model may be transferable to past-time bioclimatic conditions (Jiménez-Valverde et al., 2009).

A total of 55 occurrence records of *Nanorana pleskei* were selected from the GBIF web site (www.gbif.es) and our field records (Table S3). Spatially auto-correlated occurrence points could make models biases in SDM (Veloz, 2009; Hijmans, 2012; Boria et al., 2014). So our occurrence localities were spatially rarefied at 20 km^2^ based on topographic and climate heterogeneity (Fig. S1), resulting in 46 points for SDM (Table S3). Additionally, a latitudinal background selection bias file accounting for bias associated with latitudinal changes in the studied region and limiting selection of background points to the regions only feasibly colonized by the species was created for SDM (Brown, 2014). In this analysis, the distance to a buffer minimum convex polygon (MCP) (Kremen et al., 2008) was set as two decimal degrees.

To optimize the model performance (Shcheglovitova & Anderson, 2013), different combinations of the five model feature class types (1. linear, 2. linear and quadratic, 3. hinge, 4. linear, quadratic and hinge and 5. linear, quadratic, hinge, product and threshold, as in (Brown, 2014) and a range of regularization parameters (0.5–5; in 0.5 increments)) were tested. The distribution models were calibrated using spatial jackknifing (*k* = 3) (Brown, 2014), and the best model was chosen with the lowest omission rate and the highest AUC (the area under the receiver operating characteristic curve) (Fielding & Bell, 1997). AUC > 0.8 means that the model performed well (Gassó et al., 2012). The cross-validation strategy with 90% of the presence data as training data and the remaining 10% as test data was used to access the accuracy of models. The model used 10,000 maximum iterations and 10 replications. The 10th percentile training presence logistic threshold commonly used for models comparison (Pearson, 2007) was specified.

The SDMs of all periods were summed and reclassified for predicting stable niche regions (Chan, Brown & Yoder, 2011). To correct over-predictions (Brown, 2014), the final SDMs were clipped by a buffered MCP as above. To visualize the spatial connectivity, dispersal networks among populations were constructed based on the shared mtDNA haplotypes using the weighted least-cost-corridors method in Chan, Brown & Yoder (2011). A friction layer used for least-cost-corridors calculations was created by inverting the niche stable map (Brown, 2014).

The predictions under current and historical bioclimatic conditions were created by MAXENT, and the other operations associated with SDM were implemented in the program SDMTOOLBOX v1.1b (Brown, 2014) applied in ARCGIS.

Ancestral locations were inferred using the BI discrete areas in the software RASP v3.2 (Yu et al., 2015). MCMC trees and the final time tree resulted from the dating estimations above were used as input genealogies. Our 15 sampling locations with GPS coordinates were coded as independent characters. MCMC chains were run for five million iterations with sampling every 1,000 iterations and discarding the first 10% as burn-in. Ancestral sites with their origin ages were interpolated into a continuous map in ArcGIS.

## Results

### Phylogenetics and dating

The two-gene mtDNA dataset was with length of 1,548 bps, contained 55 variable nucleotide positions, with 44 parsimony informative sites, respectively. ML and BI analyses yielded almost consistent topology (Fig. 1B) except some tips with low supports. ML bootstrap proportions (bp) and Bayesian posterior probabilities (bpp) were more than 95% supporting the monophyly of *Nanorana pleskei*. In *Nanorana pleskei*, two major lineages (north and south) were revealed. Lineage north (bp = 71/bpp = 0.96) was comprised of two sublineages, e.g., sublineages northwestern (NW; bp = 94/bpp = 1.00) and northeastern (NE; bp = 50/bpp = 0.94), which were allopatric (Fig. 1A). Lineage south (bp = 88/bpp = 1.00) also contained two sublineages, sublineages S1 (bp = 60/bpp = 0.52) and S2 (bp = 65/bpp = 0.95), which were overlapped in two populations (population 1 and 2; see Fig. 1A).

Mean pairwise uncorrected *p*-distances within lineages north and south were 0.4% and 0.2%, respectively, much lower than that between them (2.1%). Similarly, the *p*-distance between sublineages NW and NE was 0.6%, larger than that within them (NW: 0.1%, NE: 0.2%). But the *p*-distance between sublineages S1 and S2 was 0.2%, little larger than that within them (S1: 0.1%, S2: 0.1%).

The divergence between two major lineages north and south was traced back to about 1.41 million years ago (Mya) with 95% highest posterior density (95% HPD: 2.39–0.85 Mya), and divergence time was 0.55 Mya (95% HPD: 0.78–0.21 Mya) within lineage North and 0.25 Mya (95% HPD: 0.49–0.1 Mya) within lineage south, respectively (Fig. 1B).

### Population genetic structure and demographic history

**Figure 2:**
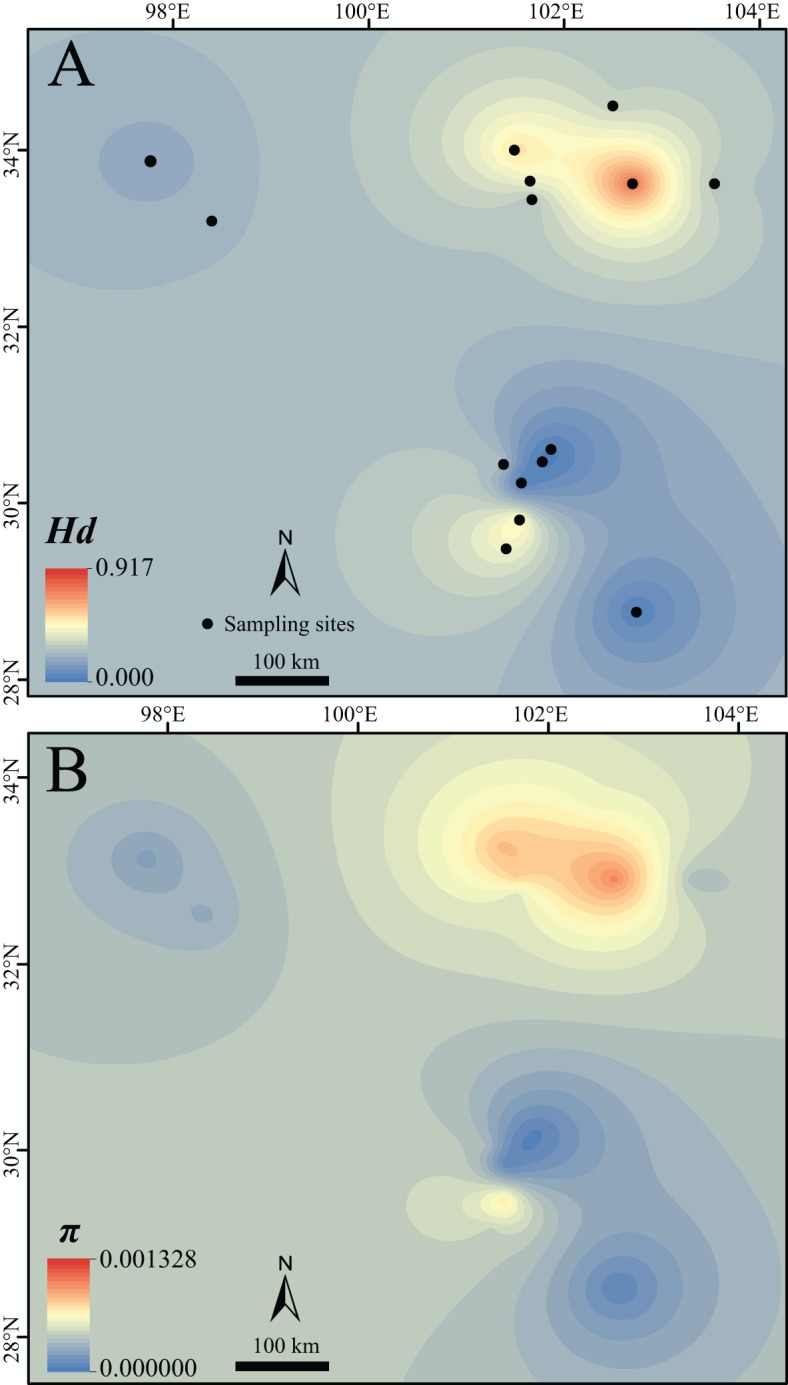
Surface of the interpolated genetic diversity of *Nanorana pleskei*. (A) *Hd*, haplotype diversity. (B) *π*, nucleotide diversity. Warmer color indicates higher genetic diversity. The interpolation was conducted under a kriging framework.

Lineage north and sublineage NE had much higher *Hd* and π than the sublineage NW, lineage South and sublineages S1 and S2 (Table 1; Fig. 2). As noted, *Hd* of sublineages S2 was quite low (Table 1).

**Table 1:** Genetic diversity, neutrality tests and mismatch goodness-of-fit tests in the mtDNA lineages.

mtDNA lineage	*n* (*nh*)	*hd* (s.d.)	*π* (s.d.)	Neutrality tests		Goodness-of-fit tests		Mismatch distribution
				Tajama’s *D*	Fu’s *F*s	SSD (*P* value)	HRI (*P* value)	
North	101 (16)	0.815 (0.022)	0.00298 (0.00014)	−0.0105	−0.3348	0.0609 (0.0848)	0.1078 (0.0485)	Multimodal
NW	33 (4)	0.278 (0.098)	0.00019 (0.00007)	−1.3876	−2.3837*	0.0058 (0.4250)	0.2720 (0.5727)	Unimodal
NE	68 (12)	0.758 (0.039)	0.00114 (0.00011)	−1.1488	−3.6383	0.0214 (0.1875)	0.0873 (0.2308)	Bimodal
South	87 (7)	0.553 (0.051)	0.00082 (0.00008)	−0.4970	−0.5345	0.4219 (0.0000)	0.2147 (0.9851)	Multimodal
S1	30 (4)	0.572 (0.052)	0.00041 (0.00006)	−0.3958	−0.6853	0.0320 (0.0477)	0.2202 (0.0388)	Bimodal
S2	57 (3)	0.070 (0.046)	0.00007 (0.00005)	−1.6818*	−2.4707**	0.0015 (0.1247)	0.8039 (0.8077)	Unimodal

**Notes:**

*n*, number of samples; *nh*, number of haplotypes; π, nucleotide diversity; *hd* (s.d.), haplotype diversity with standard deviation; SSD, sum of squared deviation between the observed and expected distribution of pairwise differences; HRI, Harpending’s raggedness index.

* Denotes significance at α = 0.05.

** Denotes significance at α = 0.01.

TCS analysis of mtDNA data yielded two allopatric and unconnected haplotype groups (95% confidence interval; Fig. 1C), corresponding to the lineages north and south (Fig. 1B). In lineage north, haplotypes from all northeastern populations were mainly clustered into three loops, and haplotypes from all the northwestern populations showed a star-like shape and connected with the northeastern populations in six steps. In lineage south, sublineage S1 had two dominant haplotypes but S2 showed star-like shape. Although the nuclear Rhod gene only had three haplotypes and did not reveal well-supported haplogroups, the northern populations had their own distinct haplotypes as well as the southern populations (Fig. 1D).

Analyses of molecular variance showed significant levels (*P* < 0.01) across all hierarchical levels when all grouping allocations were tested (Table 2), rejecting the model of no population genetic structure. Highest *F*ct (95.951%) was found when using grouping arrangement with four sublineages, but considerable *F*ct was also obtained for grouping arrangements according to two major lineages (*F*ct = 94.317%) and three geographic groups (*F*ct = 88.647%). IBDWS analyses resulted in an uncorrelated relationship (*r* = 0.2593; *P* = 0.373) between genetic distance (*F*st/1 − *F*st) and geological distance, rejecting the IBD model.

**Table 2:** Results of analysis of molecular variance (AMOVA) of mtDNA data.

Grouping arrangement	Among groups *F*ct (% of variation)	Within groups *F*sc (% of variation)	Within populations *F*st (% of variation)
North, south	88.647	83.741	98.154
NW, NE, S1, S2	95.951	39.108	97.534
NW, NE, south	94.317	61.842	97.832

**Note:**

Lineages (north and south) and sublineages (NW, NE, S1 and S2) were showed in Fig. 1.

**Figure 3:**
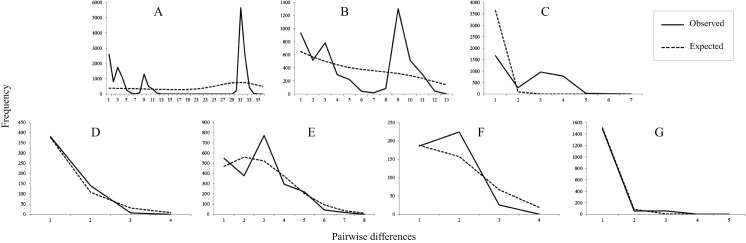
Mismatch distributions of mtDNA lineages. (A) All populations. (B) Lineage north. (C) Lineage south. (D) Sublineage NW. (E) Sublineage NE. (F) Sublineage S1. (G) Sublineage S2. The lines indicate the observed frequency of pairwise nucleotide differences between sequences, and the dashed lines are the expected distribution based on a model of sudden population expansion.

Tajima’s D value in Neutrality tests was significantly negative (*P* < 0.05) only in sublineage S2, suggesting past population expansion and significantly negative (*P* < 0.01) Fu’s FS values were found in sublineages NW and S2 (Table 1). In mismatch distribution, SSD and HRI values suggested population expansion model for the sublineages NE, NW and S2. sublineages NW and S2 exhibited an observed unimodal mismatch frequency distribution (Fig. 3), fitting a recent sudden population expansion model. The BSP for sublineages NW, S1 and S2 was not constructed because of few variations accounting for short coalescent time in each of them. BSPs (Fig. S2) depicted a model of past increase of effective population size in total population of the species (from about 0.05 Mya), lineage north (from about 0.05 Mya) and sublineage NE (from about 0.02 Mya), but this model was not revealed in lineage south suffering short coalescent time with stable population size.

### Species distribution modeling and ancestral locations

**Figure 4:**
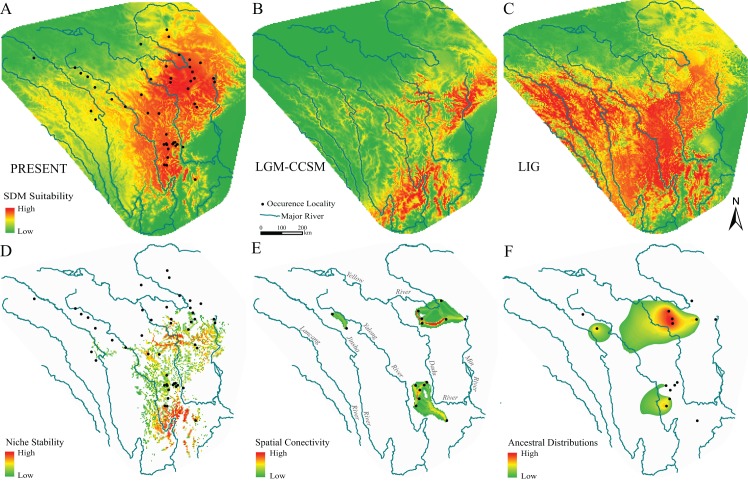
Species distribution models, hypotheses of habitats stability and spatial genetic patterns of *Nanorana pleskei*. (A–C) Species distribution models of *Nanorana pleskei* under present and paleo periods: (A) present time; (B) LGM–CCSM; (C) LIG. (D) Stability of the niche of *Nanorana pleskei* through all periods based on a species distribution model. (E) Dispersal networks of *Nanorana pleskei* depicting the connectivity of populations through suitable habitat. (F) Relative age and areas of ancestral distributions of mtDNA lineages as inferred by RASP. Warmer colors depict higher probability.

The model performances were good and robust because the mean ± standard deviation of AUC was 0.880 ± 0.007 and 0.876 ± 0.048 for the training data and test data, respectively. The SDM under present bioclimatic conditions showed substantial habitat suitability across the current known range of the amphibian species on the eastern Tibetan Plateau (Fig. 4A). The distribution predicted under MIRCO of LGM was similar to the CCSM model (Fig. 4B). Compare to the current distribution, during the LGM, the predicted distribution exhibited obvious contraction to the northeastern region, but in the northwestern region, suitable habitats still existed in the main valley of the upper-middle reaches of Jinsha and Yalong rivers (Fig. 4B). During the LIG, the predictions showed a slight south-direction shift of the distribution range (Fig. 4C). The stable suitable habitats were constructed by summing all SDMs of different periods, and were showed using the highest 30% reclassified values. The results suggested that the stable suitable habitats existed in the regions of eastern half current range of the species (Fig. 4D).

Because there were no haplotypes between three major geo-groups, spatial connectivity showed by dispersal networks between populations was constructed only within each of them (Fig. 4E). In the southern group, high connectivity was presented near the Yalong River, and in the northeastern group, the highest connectivity existed between the populations near the upper reach of Yellow River, but in the two northwestern populations, the level of connectivity was relatively low.

Ancestral geographical distributions for major mtDNA lineages were inferred to be located in the three geo-groups same as the dispersal-network analyses: region near the middle Yalong River, northeastern region near the upper Yellow River and Dadu River and northwestern region near the upper Yalong and Jinsha valleys (Fig. 4F).

## Discussion

Integrative application of phylogeographic investigation and ecological niche modelling has become a representative approach for explaining intraspecific evolutionary patterns associated with climate oscillations (Chan, Brown & Yoder, 2011; Waltari et al., 2007; Edwards, Keogh & Knowles, 2012). As a case, our study on endemic frog species *Nanorana pleskei* in the eastern Tibetan Plateau revealed concordance of genetic and ecological modelling signals: first, times of diversification of its lineages occurred through the middle-late Pleistocene, basically fitting scenarios of regionally paleo-climatic fluctuations on the Tibetan Plateau; second, deeply independent origin of major lineages and the spatial interpolations indicated multiple refugia for the species existing across the eastern Tibetan Plateau, also revealed by the niche stability of paleo and current periods across its main distribution range. The integrations and fitting-models allocated substantial insights on evolutionary history of the species and its response to climatic fluctuations.

In *Nanorana pleskei*, two major mtDNA lineages (north and south) were revealed, showing no shared haplotypes and deep allopatric divergence (*ca.* 1.41 Mya; Fig. 1B), a similar find as supported by the analyses on the nuclear Rhod gene, i.e., north and south populations had their own haplotypes (Fig. 1D). Recent uplifts of the Tibetan Plateau and Pleistocene glaciations could contribute to the isolations in modern species (Wang et al., 2010; Liu et al., 2015; Zhou et al., 2014). Accordingly, in the range of the lineage North, from *ca.* 1.2 Mya, the upstream of the Yellow River system had been dramatically upraised (named as the Kunlun-Yellow River tectonic movements; Cui, Wu & Liu, 1997), increasing the landscape differentiations between this region exhibiting open and broad prairie and the range of the lineage south carrying high topographical heterogeneity with tableland and alpine valleys at the southeastern margin of plateau (Fig. 1A; Fig. S1). The movement also promoted expansions of ice-sheets, e.g., the oldest glaciation in the Tibetan Plateau was reported beginning from *ca.* 1.17 Mya (Shi, Zheng & Su, 2006). Obviously, the early Pleistocene geological and climatic fluctuations would contribute to the isolation and deep divergence of the two lineages. This pattern has also been found in other organisms in this region (Wu et al., 2013; Guo, Liu & Wang, 2012). The divergence of sublineages NW and NE (*ca.* 0.55 Mya) was likely derived from similar historical factors. Continuous extension of the Kunlun-Yellow River tectonic movement during the middle Pleistocene had promoted the differentiation between hinterland of Tibetan Plateau and edge regions. The range of northwestern lineage (NW) was west of the 600 mm annual precipitation, and adjoined platform of Tibetan Plateau, being more arid and drier, in favor of maintaining its genetic independence from eastern regions. Of course, the maximum glaciation occurred during *ca.* 0.77–0.5 Mya (Shi, Zheng & Su, 2006) might also provide climate-driving force on separation of it from eastern lineages.

In this view, the species had been likely derived from multiple glacial refugia rather than single refugium. What is more, several lines of evidence support the scenario of multiple glacial refugia at least on the eastern plateau. Firstly, the single-refugium hypothesis generally advocated star-like haplotype network in the whole population and presented decreasing trends on genetic diversity from one single refugium to the newly colonized routes and/or fringe areas (Provan & Bennett, 2008). The pattern was not revealed in *Nanorana pleskei*. Not only mtDNA data but also nuclear DNA data presented two major distinct haplotype groups (Figs. 1C and 1D), especially in mtDNA, the two lineages independently possessed high genetic diversity (Table 1). As mentioned above, these two lineages dated back to the early Pleistocene much earlier than the LGM were allopatric, implying that they had probably occupied independent glacial refuges. Secondly, the regions with stable suitable environments through history were often inferred as the glacial refugia (Chan, Brown & Yoder, 2011). Our SDMs of current and paleo periods (LIG and LGM) indicated that stable suitable habitats through times for the species existed in almost half of its current distributed range mainly in the basins of major rivers (Fig. 4D). Even in the northern and western range, major valleys, e.g., upper-middle reaches of Yellow River, Dadu River, Yalong River and Jinsha River, still hosted considerable niche stability for the frog (Fig. 4D). Ancestral area distribution also inferred that the MRCA of the populations in these areas were relative old (Fig. 4F). Thus, it is therefore plausible to postulate that these regions had been occupied as the refugia for different lineages. Some other plateau species have likely also taken refuge in the regions (Liu et al., 2013; Zhou et al., 2013). Finally, the multiple-glacial-refugia scenario fits well with the multi-regions and multi-scales glaciations hypotheses during the Pleistocene (Owen et al., 2005; Yang et al., 2008). Moreover, substantial proofs indicated that many districts (e.g., Jinsha River, Yalong River and northeastern region) in the range of *Nanorana pleskei* were free of ice even in mid-Pleistocene glacial periods on Tibetan plateau (Shi, Zheng & Su, 2006).

Distinct refugia environments would generally create different characteristics of demographic dynamics for the separated lineages (Avise, 2000). For lineage south, the neutrality tests and mismatch distributions all did not show signals of a recent expansion model (Table 1), and BSP showed stable population size through short coalescent time (Fig. S2). But its sublineage S2 significantly fitted the recent expansion model (Table 1), and distributed from central of genetic-diversity map to the edge populations (Fig. 2). This indicated that the lineage south has been probably experienced bottleneck effects, but its sublineage S2 has been probably experienced founder effects and a recent expansion (Zink, 2002). A little is similar for the sublineage NW, the relative older ancestor of it with low genetic diversity and a recent sudden expansion model indicated possible historical bottleneck effects in it (Zink, 2002). However, for the lineages NE possessing high genetic diversity, BSPs showed population increase even through the LGM (Fig. S2) but Fu’s Fs and Tajima’s D did not support the model (Table 1). The interpretation for this discordance probably indicated that the populations underwent expansion but then were recently subdivided, subjected to substantial migration, and/or had experienced historical contractions (Ray, Currat & Excoffier, 2003; Castoe, Spencer & Parkinson, 2007; Bertorelle & Slatkin, 1995).

Of course, there are reasons to be cautious about the findings. In this study, only gene trees based on mtDNA were used to estimate the divergence times and the estimated age of differentiation of *Nanorana pleskei* lineages predated the time frame used for the SDMs. Compared with species tree methods based on multiple loci, the gene tree method tends to overestimate the divergence times (McCormack et al., 2010). Even though it is true for the findings that the history of major lineages was much probably earlier than the LGM, there still were some other models of demographic patterns, for example, populations had probably been experienced contractions and expansions several times due to glacial cycles during Pleistocene. To now, in view of no fossil information and “incomplete” sampling, we cannot appraise the veracity of estimations on divergence times. So in the future work, “complete” sampling and inclusion of species tree method based on multiple loci even genomic data may be the guarantee to verify the models.

The novel phylogeographic pattern provided important conservation implications for the frog species. The long-term allopatric lineages with limited spatial connectivity (Fig. 4E) should be recognized as independent evolutionarily significant units (ESUs) for future protection (Crandall et al., 2000). The drainage basins in the range of the species hosted important water source for the Yellow River and Yangtze River systems and were indicated as the refugia for it. These valleys have been prominently disturbed by human activity especially in the new century. Thus, more effective measures and managements are desired for protecting habitats in the refuges.

## Conclusion

This study integrating phylogeographic investigation and ecological niche modelling suggested that Pleistocene climatic fluctuations combined with the uplifts of Tibetan Plateau to profoundly impacted the evolutionary and distributional patterns of the plateau endemic amphibian species. *Nanorana pleskei* lineages have taken refuge in upper-middle streams of the Yellow River, Dadu River, Jinsha River and Yalong River because the regions have probably loaded stable, long-term suitable habitats. Multiple refugia environments have probably promoted the lineages possessing distinct demographic characteristics. These points provide useful conservation implications for the species.

## Supplemental Information

10.7717/peerj.3770/supp-1 Supplemental Information 1 Genetic diversity and results of Neutrality tests and Mismatch distribution analyses for each MtDNA lineages.

10.7717/peerj.3770/supp-2 Supplemental Information 2 Primers designed in this study for amplifying and sequencing COI and ND1 genes.

10.7717/peerj.3770/supp-3 Supplemental Information 3 Occurrence data for species distribution modelling.

10.7717/peerj.3770/supp-4 Supplemental Information 4 Climatic and topographic heterogeneity in the eastern Tibetan Plateau. (A) Climatic heterogeneity. (B) Topographic heterogeneity. Warmer color means higher heterogeneity. (C) A landscape photo of the northern region, showing open landscape in prairie. (D) A landscape photo of the southern region, showing narrow landscape in mountains and valleys landscape.

10.7717/peerj.3770/supp-5 Supplemental Information 5 Bayesian Skyline plots for some lineages of *N. pleskei*. The bold black line indicates the median value of effective population size; the thin black lines denote the 95% highest posterior probability interval. The y-axis correspond to population size Ne* Tau (effective population time x generation length time in millions of years).

10.7717/peerj.3770/supp-6 Supplemental Information 6 Raw data sequences.

## Additional Information and Declarations

### Field Study Permissions

The following information was supplied relating to field study approvals (i.e., approving body and any reference numbers): Field experiments were approved by the Management Office of the Zoige Nature Reserve (project number: ZNR201303006).

### DNA Deposition

The following information was supplied regarding the deposition of DNA sequences: The sequences described here are accessible via GenBank accession numbers: KX806663, KX806664–KX806851, KX806852–KX807039, KX807040–KX807069, KX807070, KX807071–KX807072. We have also uploaded the sequences in a Supplemental File.

### Data Availability

The following information was supplied regarding data availability: The raw data has been supplied as Supplemental Dataset Files.
