# Supplementary figures and images for: Phylogeographic investigation and ecological niche modelling of the endemic frog species Nanorana pleskei revealed multiple refugia in the eastern Tibetan Plateau
Source: PeerJ. 2017 Sep 11;5:e3770. doi: 10.7717/peerj.3770 (PMC5598431; doi:10.7717/peerj.3770)

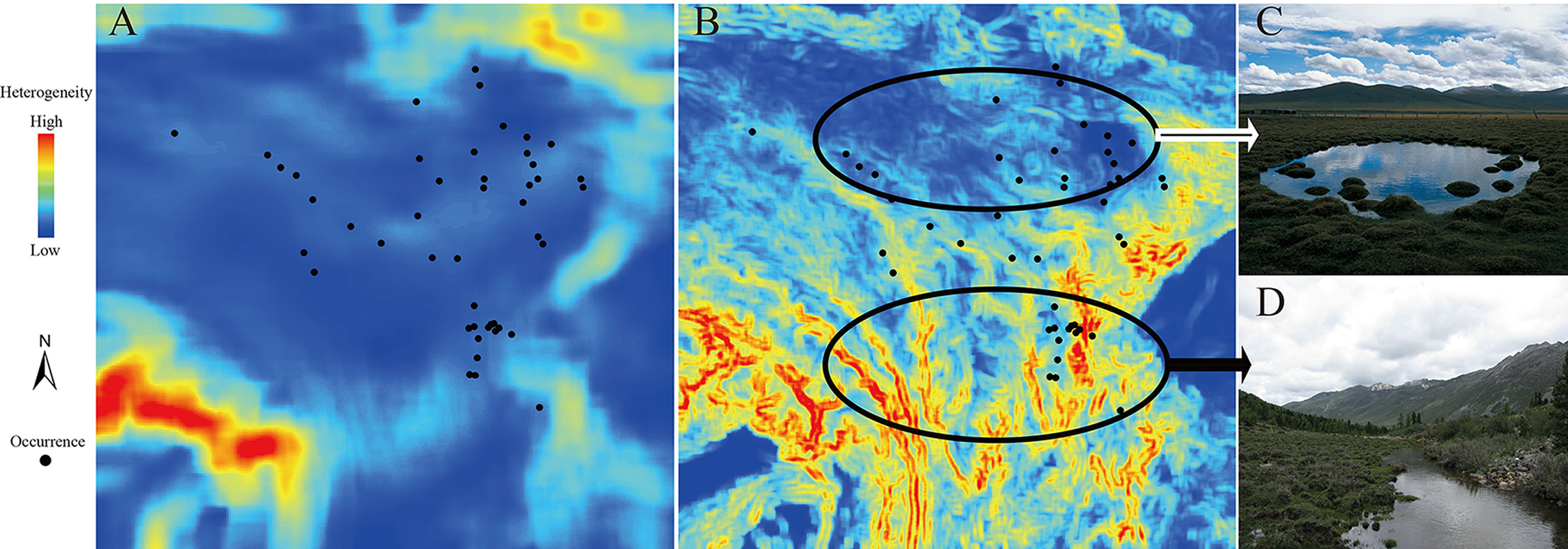

Supplement: Supplemental Information 4 — (A) Climatic heterogeneity. (B) Topographic heterogeneity. Warmer color means higher heterogeneity. (C) A landscape photo of the northern region, showing open landscape in prairie. (D) A landscape photo of the southern region, showing narrow landscape in mountains and valleys landscape. [file peerj-05-3770-s004.jpg]

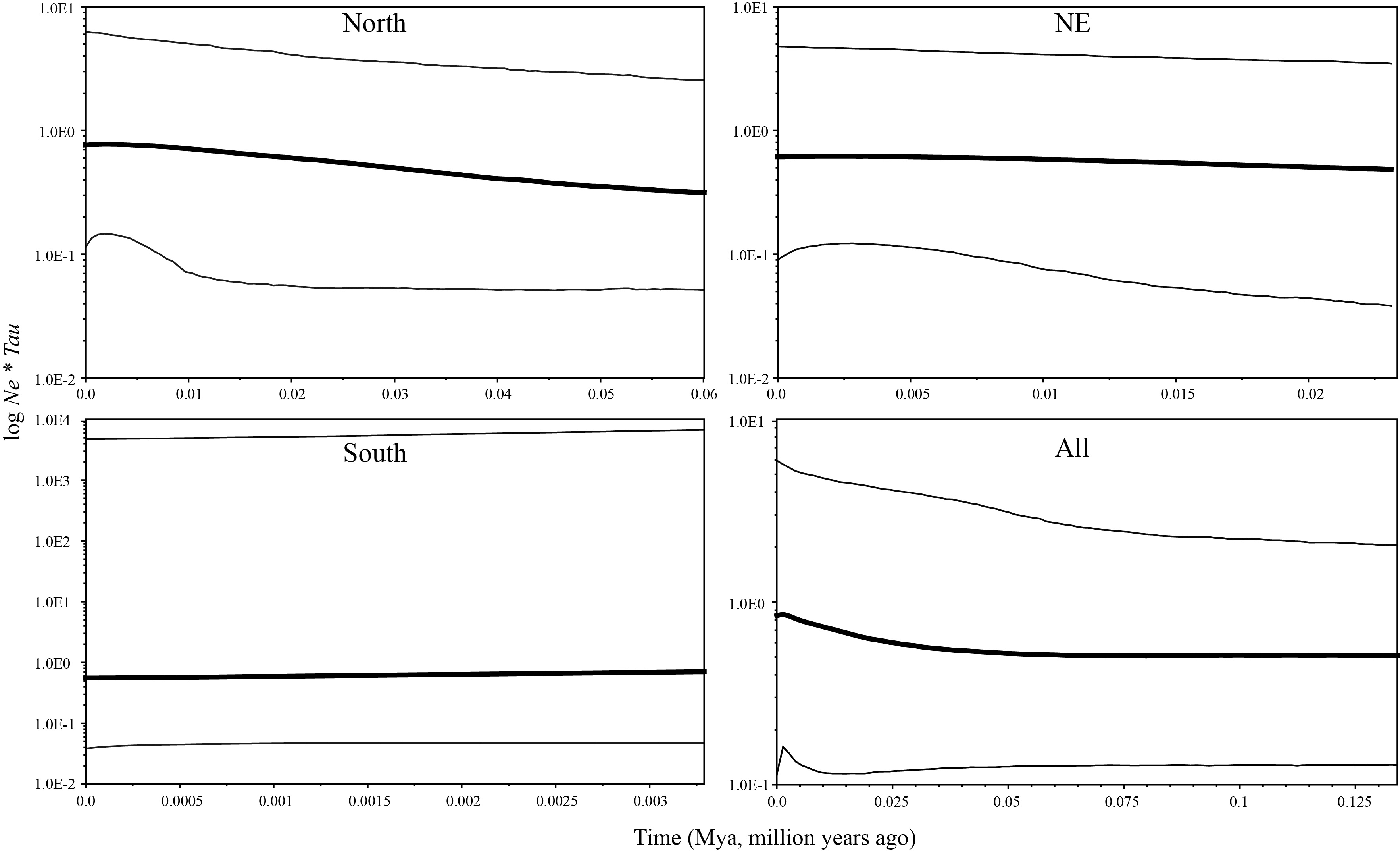

Supplement: Supplemental Information 5 — The bold black line indicates the median value of effective population size; the thin black lines denote the 95% highest posterior probability interval. The y-axis correspond to population size Ne* Tau (effective population time x generation length time in millions of years). [file peerj-05-3770-s005.jpg]
